# A Prospective Follow-Up Study on the Disease Course and Predictors of Poor Outcomes in a Random Population-Based Cohort of Newly Diagnosed Lupus Patients

**DOI:** 10.7759/cureus.27430

**Published:** 2022-07-29

**Authors:** Saadat Ullah, Muhammad Haroon, Farzana Hashmi, Zaid Tayyab, Saba Javed

**Affiliations:** 1 Division of Rheumatology, Fatima Memorial Hospital College of Medicine and Dentistry, Lahore, PAK

**Keywords:** education status, haq, hematologic manifestations, stress, sle

## Abstract

Background and objective

Systemic lupus erythematosus (SLE) is one of the most devastating systemic autoimmune connective tissue diseases. There is a paucity of prospective data on Pakistani SLE patients, and in this prospective study, we aimed to investigate the disease course, clinical outcomes, and the predictors of poor outcomes in a random population-based cohort of newly diagnosed SLE patients (diagnosed within the last one year).

Methods

This was a prospective observational study carried out in the rheumatology department of the Fatima Memorial Hospital, Lahore. Lupus patients are regularly reviewed in our dedicated lupus clinic every one to three months. For the purpose of this study, a focus group of newly diagnosed patients (diagnosed within the last one year) attending our lupus clinic was identified and prospectively followed up for 12 months. A wide range of demographical and clinical parameters was recorded. The association of clinical variables with the progressive disease was determined using univariate and multivariate logistic regressions.

Results

Prospective data of 89 newly diagnosed SLE patients regularly attending our dedicated lupus clinic were reviewed. During the study period, (January 2021 through January 2022), these patients had multiple visits overall - median: five, minimum: three, and maximum: nine visits [interquartile range (IQR) 4-7]. All 89 patients had completed one year of follow-up. Of note, 46% of the cohort was noted to have an ongoing active disease during the majority of visits in the study period. On multiple logistic regression analysis, there was a significant association between ongoing active disease ("progressors") and low education status [odds ratio (OR): 2.81, 95% confidence interval (CI): 1.01-7.76, p=0.046], stress at home (OR: 5.8, 95% CI: 2.13-15.8, p=0.001), and hematologic manifestations (OR: 3.0, 95% CI: 1.08-8.32, p=0.03).

Conclusions

Almost half of our cohort of lupus patients demonstrated active disease manifestations throughout the one-year prospective follow-up, and these were found to be associated with low education status, stress at home, and hematological manifestations.

## Introduction

Systemic lupus erythematosus (SLE) is one of the most devastating autoimmune connective diseases, where multisystem involvement ranges from cutaneous to neurological manifestations along with highly unpredictable patterns of remissions and flares. Evidence to date largely favors the role of genetic and environmental factors in its development. Significant racial differences have been reported across the globe with regard to its prevalence, organ involvement, and outcomes [1].

SLE, similar to other autoimmune diseases, is associated with many prognostic factors, such as age, sex, ethnic origin, socioeconomic class, and genetics, and it is a chronic illness [2,3]. Certain poor prognostic indicators of SLE are associated with a much higher prevalence and incidence of the disease [4]. Although SLE affects people of all ages, it has been observed with more severe symptoms such as malar rash in patients of younger age [3]. As opposed to age, the gender predilection [3] of SLE is very pronounced, and it affects females at a significantly higher rate than males (with a female-to-male ratio of 9:1) [5]. Ethnic origin plays a role in the prognosis of SLE; for instance, it is observed in more severe forms among blacks, but poor socioeconomic status needs to be ruled out in this specific context [6]. Moreover, a lower socioeconomic status itself is a great hindrance to a better quality of life and health in patients with SLE irrespective of their access to quality healthcare [7].

Apart from the demographic variables describes above, there are other factors that are responsible for poor outcomes in SLE [8]. The prognosis of SLE varies greatly from patient to patient, and the disease progression is closely linked to the general health of the individual patient [9,10]. Associated with several poor prognostic factors, SLE is a disease with significant mortality rates all over the world [11]. Certain indicators of a poor standard of living, such as poor literacy [12], and low ratings pertaining to several factors included in the Health Assessment Questionnaire (HAQ) regarding routine activities of living, i.e., dressing, getting up, eating, walking, grip, etc. [13] are significant poor prognostic factors in terms of recovery from SLE. Renal involvement in SLE is considered the most influential or crucial aspect responsible for a poor prognosis [14]. Shrinking lung syndrome also contributes to a poor prognosis in SLE in addition to the involvement of the renal system to a large extent [15]. Among patients of SLE with hematological disorders, the presence of catastrophic antiphospholipid syndrome (CAPS) is another clear indication of a poor prognosis [16]. Stress, hospitalization, and other social stigmas with regard to cosmetic reasons also contribute significantly to poor outcomes in SLE [17]. There are several poor prognostic indicators documented in terms of the response to the treatment for SLE [18]. In this prospective study, we aimed to investigate the disease course, clinical outcomes, and predictors of poor outcomes in a random population-based cohort of SLE patients.

## Materials and methods

Study design and setting

This was a prospective observational study carried out in the rheumatology department of the Fatima Memorial Hospital, Lahore, which is the leading tertiary-care rheumatology unit in the country. A dedicated lupus clinic is being run in our department for nearly a decade. Lupus patients are regularly reviewed in this clinic every one to three months, depending on their clinical needs, and at each visit, the validated SLEDAI-2K index is measured and documented. For the purpose of this study, a focus group of newly diagnosed (diagnosed within the last one year) patients attending our lupus clinic was identified and prospectively followed up for 12 months. All studied patients had completed one year of follow-up. Patients' data from three visits of almost equally spaced intervals during this one-year follow-up at our lupus clinic was recorded. Institutional review board approval and informed consent were obtained (FMH-10-2020-IRB-827-M).

Study variables and data collection

The clinical variables studied were gender, age, smoking habits, body mass index, education status, marital status, type of residence (rural/urban), disease duration, and functional abilities of the cohort as assessed by the Health Assessment Questionnaire (HAQ). Different organ involvements were recorded. Those patients who had an ongoing active disease during most of their recorded visits or had developed new organ involvement were labeled as "progressors". Patients were stratified by education status based on whether they had completed secondary (high) school education. Evaluation of disease activity and severity was made as per internationally recognized and validated measures, and for this study, we extracted data from routinely collected SLEDAI-2K activity measures. All participants were directly queried via an interview at the time of patient enrolment about the presence or otherwise of mental/emotional stress at home, and asked to rate their responses on a scale of 1-3 (mild, moderate, and severe) [19]. For better understanding and ease of data analysis, a dichotomous variable was constructed with moderate-to-severe stress patients categorized into one group and non-to-mild stress patients into another group. A sample size of 87 SLE patients was determined based on a 90% confidence interval (CI), 5% level of significance, and 3% margin of error by assuming a lupus prevalence of 30 per 100,000 population in Asia [20].

Statistical analysis

Statistical analysis was performed using SPSS Statistics version 25.0 (IBM Corp., Armonk, NY). A p-value <0.05 was considered statistically significant (two-tailed). An χ2 statistic was used to investigate the distributions of categorical variables, and continuous variables were analyzed using the Student's t-test. We applied odds ratio (ORs) and associated confidence interval (CI) to measure the association between different variables. The association of clinical variables with the progressive disease was determined using univariate and multivariate logistic regressions. The factors on univariate analysis with significance at the 0.25 level were entered into a multivariable model. The model was then reduced by backward elimination until the remaining effects were significant at the 0.05 level. Estimates of regression coefficients were obtained from this final model.

## Results

A total of 237 Lupus patients are regularly followed up in our rheumatology department, and among them, 112 patients were deemed eligible for this study given that they had been diagnosed with lupus in the preceding year. Based on our target sample size, 89 patients fulfilling the European League Against Rheumatism (EULAR)/American College of Rheumatology (ACR) 2019 classification criteria [21] were recruited. The prospective data of these 89 newly diagnosed SLE patients regularly attending our dedicated lupus clinic were reviewed. During the study period (January 2021 through January 2022), these patients had multiple visits overall - median: five, minimum: three, and maximum: nine visits [interquartile range (IQR): 4-7]. All 89 patients enrolled were followed up for 12 months. Table 1 summarizes the characteristics of the cohort. The mean age of the cohort was 25 ±6 years, 92% were female, the mean disease duration was 12.4 ±11 months, 41.6% of the cohort were married, 26% resided in rural areas, 38% had a low education status, and 42.7% had moderate-severe stress. Regarding different organ manifestations of SLE, 86.5% of the cohort was noted to have mucocutaneous disease, 37% had hematological manifestations, and 45% had renal involvement. Of note, 46% of the cohort was noted to have an ongoing active disease during the majority of visits in the study period (termed as progressors in this study), and 14.6% of the cohort developed new organ involvement during the study period.

**Table 1:** Characteristics of the studied cohort of lupus patients SD: standard deviation; HAQ: Health Assessment Questionnaire

Variables	Values
Age, years, mean ±SD	25 ±6
Female gender, n (%)	82 (92)
Disease symptom duration, months, mean ±SD	12.4 ±11
Marital status: unmarried, n (%)	37 (41.6)
Rural residence, n (%)	23 (26)
Low education status: ≤primary school, n (%)	34 (38)
Progressors, n (%)	41 (46)
New organ involvement, n (%)	13 (14.6)
Moderate-severe stress, n (%)	38 (42.7)
Mucocutaneous disease, n (%)	77 (86.5)
Hematological involvement, n (%)	33 (37)
Renal involvement, n (%)	40 (45)
HAQ score, mean ±SD	0.86 ±0.56

As displayed in Table 2, compared to those with their disease in remission, patients with ongoing active disease (progressors) had significantly more stress (p<0.001), low education status (borderline significance of p=0.08), significantly worse functional capacity as per HAQ scores (p<0.001), and more hematological and renal involvements (p<0.001). On multiple logistic regression analysis (Table 3), a significant association of ongoing active disease (progressors) was noted with low education status (OR: 2.81, 95% CI: 1.01-7.76, p=0.046), stress (OR: 5.8, 95% CI: 2.13-15.8, p=0.001), and hematological manifestations (OR: 3.0, 95% CI: 1.08-8.32, p=0.03). The following variables were included in the final regression analysis: low education, stress, predominant mucocutaneous disease, predominant renal disease, and predominant hematological disease.

**Table 2:** Comparison between progressors and the rest of the cohort SD: standard deviation; HAQ: Health Assessment Questionnaire

Variables	Ongoing active disease (progressors)	Disease in remission	P-value
Age, years, mean ±SD	25.3 ±6.6	25.7 ±5.4	0.76
HAQ score, mean ±SD	1.17 ±0.60	0.61 ±0.35	<0.001
Disease duration, months, mean ±SD	12 ±10	12.5 ±11	0.94
Female gender, n (%)	37 (90)	45 (93.7)	0.69
Married, n (%)	24 (58.5)	28 (58.3)	1.0
Rural residence, n (%)	12 (29)	12 (25)	0.81
Low education, n (%)	20 (48.6)	14 (29)	0.08
Moderate-severe stress, n (%)	27 (65.8)	11 (23)	<0.001
Mucocutaneous disease, n (%)	37 (90)	40 (83)	0.37
Hematological disease, n (%)	22 (53.6)	11 (23)	0.004
Renal disease, n (%)	26 (63.4)	14 (29)	0.001

**Table 3:** Univariate and Multivariate regression model revealing significant associations of different clinical variables with ongoing active lupus disease Variables included in the final regression analysis were as follows: low education, stress, predominant mucocutaneous disease, predominant renal disease, predominant hematological disease, and HAQ scores OR: odds ratio: CI: confidence interval; HAQ: Health Assessment Questionnaire

Variables	Univariate model			Multivariate model		
	OR	95% CI	P-value	OR	95% CI	P-value
Female gender, n (%)	1.6	0.34-7.7	0.54			
Age	0.99	0.92-1.06	0.76			
Marital status: married	1.00	0.43-2.3	0.98			
Rural residence	1.13	0.43-2.9	0.79			
Low education status	2.3	0.96-5.5	0.060	2.81	1.01-7.76	0.046
Disease duration	0.99	0.99-1.00	0.59			
Age of onset	0.99	0.93-1.05	0.80			
Moderate-severe stress	6.48	2.55-16.4	<0.001	5.81	2.13-15.8	0.001
Predominant mucocutaneous disease	1.85	0.51-6.65	0.34			
Predominant hematological disease	3.89	1.56-9.68	0.003	3.00	1.08-8.32	0.03
Predominant renal disease	4.21	1.73-10.2	0.002			
HAQ score	3.31	1.33-8.22	0.1			

## Discussion

There is a paucity of prospective data on Pakistani SLE patients, especially pertaining to the course of the disease and identifying the predictors of outcome. The current study revealed a statistically significant association of low education status, stress, and hematological manifestations with poorly controlled disease among patients with SLE.

Our study revealed that 38% of SLE patients attending our lupus clinic had low education status, and after controlling for confounders, it was found to be independently associated with poor disease control. The results of the study are similar to those of Ward and Studenski [2]. Other studies have also shown an association between poor clinical outcomes and low literacy levels, supporting the results of our study [22,23]. However, another study revealed that education had no significant role in terms of outcomes and considered it a poor prognostic factor [24]. Furthermore, it has been established that in families with low socioeconomic status coupled with low literacy or those with low education status, the risk for the disease is several folds higher compared to those with higher education status [25]. In the developed world, where other factors do play a role in this regard, it was found that disease progression and severity were much lower in patients of SLE who are well-educated, which was attributed to their awareness and knowledge owing to their high education levels [26].

The current study revealed that nearly 43% of the studied SLE patients experienced moderate to severe stress at home, and this was found to be independently associated with poorly controlled lupus disease. The study results are in line with those of Da Costa et al. who reported that psychosocial stress, negative life events, mood changes, and depression were significant poor prognostic factors [24]. Another study revealed that anxiousness, exacerbation of pain, and other functional disabilities among patients with SLE resulted in poor outcomes [27]. Psychosocial stress in all age groups irrespective of gender serves as a great risk factor for poor outcomes from SLE [28]. A recent study revealed stress as the strongest poor prognostic indicator of poorly controlled SLE [29]. Psychosocial stress single-handedly leads to a poor prognosis with flares of high intensity in activities of daily life [30]. Scientific evidence to support this has been established in a study where the response was recorded among those without daily stress versus those facing acute daily stress, and the results showed that stress greatly affected patient outcomes from SLE, and this was also true in terms of the response to similar therapy between the groups [31]. Considering other comorbidities such as COVID-19 as an indicator of psychosocial stress, it was found that irrespective of any other demographic variable, stress contributes massively to poor prognosis among patients of SLE [32]. We have not encountered a single study contradicting these findings or ruling stress out as a poor prognostic indicator. Stress at home has been identified as a major risk factor for non-compliance, and this possibly explains its indirect contribution to poor outcomes [19].

Hematological manifestations were found in 37% of our study cohort, and they were independently associated with poorly controlled lupus disease. Similar results have been reported by Hervier et al. [33]. It has been reported that prognosis is severely hampered and survival outcomes are greatly compromised in patients with SLE having hematological manifestations [34,35]. In another study, hematological manifestations, especially thrombocytopenia, have been reported to be associated with poor outcomes in terms of recovery from SLE [36]. These hematological manifestations have also been reported to be the most common observations in SLE, and they are linked to the worst prognosis of the disease and are associated with disease progression more than any other factor [37]. Similarly, another study has revealed that hematological manifestations and renal and multi-organ failure, irrespective of race, ethnic origin, and other socioeconomic disparities, are among the most common poor prognostic factors in SLE patients [38]. In one study, the survival rate was found to be alarmingly low among SLE patients who have hematological disorders such as autoimmune hemolytic anemia; this study was carried out among a nationwide cohort with a long follow-up period of 37 years [39].

The strengths of our study are as follows: 1) our study design and pattern make this study the first of its kind to be carried out among the local population of the country; however, comparing and contrasting data with those from other countries is beyond the scope of this report, and we had time limitations due to competing engagements with other research projects; 2) the study included SLE patients from all walks of life without any discrimination and irrespective of socioeconomic status, race, and ethnic origin; 3) the study focused on as many factors as possible with regard to their impact on disease outcomes, including socioeconomic class, stress at home, functional limitations, quality of life, and an extensive list of comorbidities. We acknowledge that there are some limitations to our study. Firstly, different socioeconomic classes could have been further classified, and the disease course could have been assessed separately. This would have allowed us to engage in a comparison between two different groups, i.e., upper class and lower or middle class, which would have led to a better understanding of the disease outcomes in terms of the socioeconomic status of the cohort. Another limitation of this study is the lack of information about long-term stress levels in our patients. If such data had been collected, it might have contributed to gaining a better understanding of the relationship between the progression of SLE and stress.

## Conclusions

Almost half of the lupus patients in our cohort were found to have active disease manifestations throughout the follow-up period, and these were significantly associated with low education status, stress at home, and hematological manifestations. The impact of domestic stress on SLE outcomes cannot be underestimated, and the development of prevention strategies and interventions to manage stress levels in vulnerable populations should be considered an important part of the routine management plan.
